# Green tea catechins are potent anti-oxidants that ameliorate sodium iodate-induced retinal degeneration in rats

**DOI:** 10.1038/srep29546

**Published:** 2016-07-07

**Authors:** Yaping Yang, Yong Jie Qin, Yolanda W. Y. Yip, Kwok Ping Chan, Kai On Chu, Wai Kit Chu, Tsz Kin Ng, Chi Pui Pang, Sun On Chan

**Affiliations:** 1 Department of Ophthalmology & Visual Sciences, The Chinese University of Hong Kong, Hong Kong; 2 Department of Ophthalmology, Eye and ENT Hospital of Fudan University, Shanghai, China; 3 Department of Obstetrics & Gynaecology, The Chinese University of Hong Kong, Hong Kong; 4 School of Biomedical Sciences, Faculty of Medicine, The Chinese University of Hong Kong, Hong Kong

## Abstract

Green tea extracts exhibit anti-oxidative and anti-inflammatory actions in different disease conditions. We hypothesized that green tea extract and its catechin constituents ameliorate sodium iodate-induced retinal degeneration in rats by counteracting oxidative stress. In this study, adult Sprague-Dawley rats were intravenously injected with a single dose of sodium iodate. Green tea extract (GTE; Theaphenon-E) or combinations of its catechin constituents, including (−)-epigallocatechin gallate (EGCG), were administered intra-gastrically before injection. Live imaging analysis using confocal scanning laser ophthalmoscopy and spectral-domain optical coherence tomography showed a progressive increase of degenerating profile across the retinal surface and decrease in thickness of outer nuclear layer (ONL) at Day-14 of post-injection. These lesions were significantly ameliorated by Theaphenon-E and catechin combinations with EGCG. Catechins with exclusion of EGCG did not show obvious protective effect. Histological analyses confirmed that Theaphenon-E and catechins containing EGCG protect the retina by reducing ONL disruption. Retinal protective effects were associated with reduced expression of superoxide dismutase, glutathione peroxidase and caspase-3, and suppression of 8-iso-Prostaglandin F_2α_ generation in the retina. In summary, GTE and its catechin constituents are potent anti-oxidants that offer neuroprotection to the outer retinal degeneration after sodium iodate insult, among which EGCG is the most active constituent.

Age-related macular degeneration (AMD) is a leading cause of irreversible visual impairment and blindness in most developed countries, affecting 50 million elderlies worldwide^1^,^2^. It is a progressive neurodegenerative disease affecting the macula and resulting in a significant loss of central vision in advanced stages. Advanced stages of AMD can be classified into dry and wet forms, represented by their respective clinical hallmarks of geographic atrophy and choroidal neovascularization^3^. In geographic atrophy, the pathological characteristics are atrophy of choriocapillaries, disruption of associated retinal pigment epithelium (RPE) and derangement of underlying photoreceptors^4^. RPE plays a multitude of important biological roles in regeneration of bleached visual pigments, formation and maintenance of interphotoreceptor matrix and Bruch membrane, selective transport of fluids and nutrients between photoreceptors and choriocapillaries, and phagocytosis of photoreceptors^5^. Lipofuscin, the residual bodies of phagolysosomes, accumulates within the cytoplasm of RPE cells along aging^6^. It induces oxidative stress in RPE cells since lipofuscin, when exposed to light and high oxygen tension, initiates the production of reactive oxygen species (ROS)^7^.

Clinically effective therapy is limited to neovascular AMD by repeated intravitreal injection of anti-vascular endothelial growth factor (VEGF) agents. However, there is still no efficient regimen for early AMD and geographic atrophy. Green tea extract (GTE) has been extensively shown to be anti-oxidative, anti-inflammatory and anti-angiogenic^8^, and has been proposed for treatment and prevention of cancers, cardiac diseases, obesity, diabetes, and neurodegenerative diseases. There are eight principal types of catechins identified from green tea. The major ones are (−)-epicatechin (EC), (−)-epigallocatechin (EGC), (−)-epicatechin gallate (ECG) and (−)-epigallocatechin gallate (EGCG). Among them, EGCG is the most abundant and potent antioxidant^9^. *In vitro*, in a cell culture model, EGCG reduces UVB-induced apoptosis in ARPE19 cells via oxidative stress and the JNK1/c-Jun pathway^10^. *In vivo*, in an animal model of light- and sodium nitroprusside-induced photoreceptor degeneration, EGCG protects photoreceptor cells from degeneration^11^,^12^. Other forms, including (+)-catechin (C), (+)-gallocatechin (GC), (−)-catechin gallate (CG), (−)-gallocatechin gallate (GCG), are present only in small quantities.

We previously showed that sodium iodate is a retinotoxin which selectively damages RPE by oxidative stress and disrupts the photoreceptor layer^13^. Sodium iodate-induced retinal degeneration in rats has been used as an animal model for drug/treatment testing, including stem cells and growth factors^14^,^15^,^16^. However, the treatment effects of GTE and its constituents have not been studied. Here, we hypothesized that GTE and its catechin constituents ameliorate sodium iodate-induced retinal degeneration in rats by counteracting oxidative stress induced by sodium iodate. To address this hypothesis, we determined the treatment effects of oral intake of GTE or combinations of catechins on progressive RPE and photoreceptor degeneration induced by sodium iodate in rats, using live imaging techniques including confocal scanning laser ophthalmoscopy (cSLO) and spectral-domain optical coherence tomography (OCT). The biochemical and histological effects of catechin treatments on the levels of oxidative stress markers were also evaluated.

## Results

### Longitudinal *in vivo* imaging of sodium iodate-induced retinal degeneration with green tea catechin treatments

**Figure 1:**
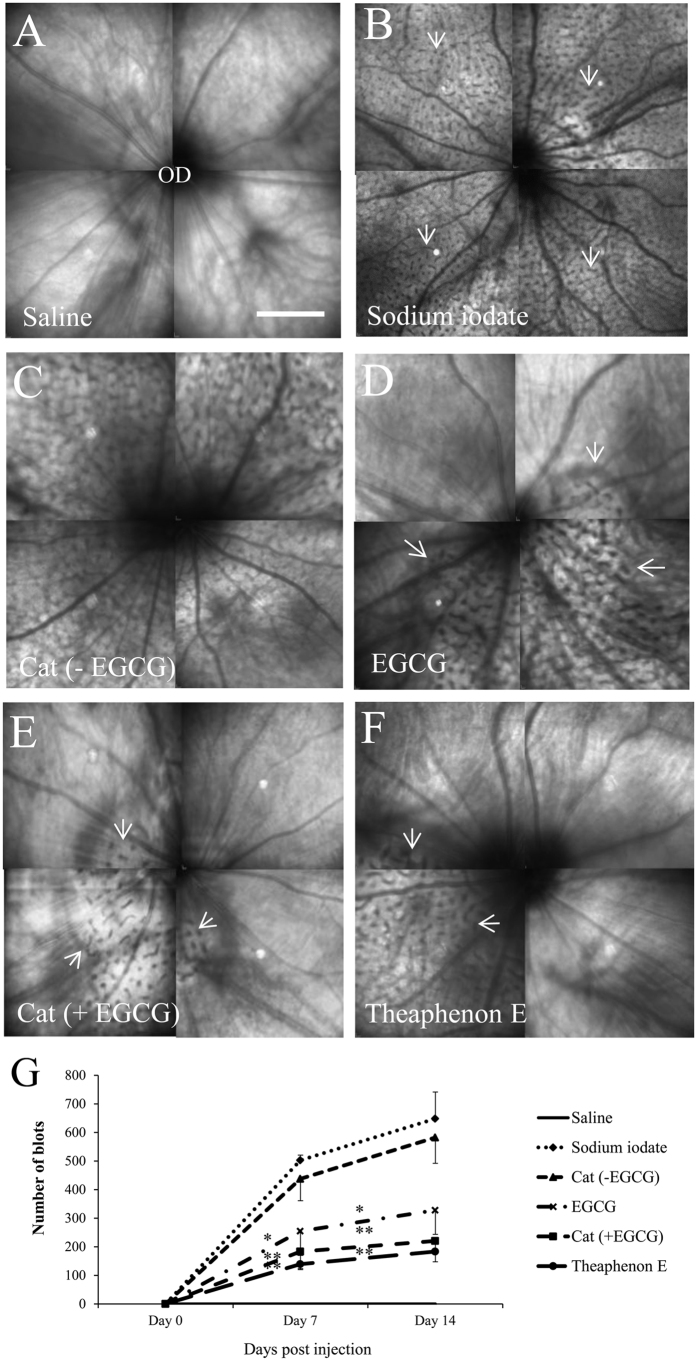
Retinal lesions under infra-red confocal scanning laser ophthalmoscopy (cSLO). Montages of fundus images showing degenerating blots in the retina of adult rats 14 days after injection of sodium iodate. (**A**) Saline control. (**B**) Dark blots (white arrows) corresponding to degenerating profiles in outer retinal layers were observed throughout the retinal surface after sodium iodate injection. (**C**) Treatment with catechin constituents (GC, EGC and EC) without EGCG [Cat (-EGCG)] did not show any reduction in the dark blots. (**D**–**F**) The dark blots were highly reduced and restricted to small regions (boundaries included by white arrows) in the retina for the groups pre-treated with GTE Theaphenon E, combination of its catechins containing EGCG. OD: optic disc. Scale bar is 200 μm. (**G**) The numbers of dark blots in cSLO images were increased progressively within 14 days after sodium iodate injection. These increases were significantly suppressed by treatments with Theaphenon E, catechins with EGCG or EGCG alone, but was not obvious with catechin without EGCG. The experimental data were compared with that of the sodium iodate group using Wilcoxon signed rank test, *p < 0.05, **p < 0.01. Data were presented as mean ± SEM.

Fundus examination by cSLO showed the typical appearance of the retinal vessels and the optic nerve head. No obvious change was visible in the retina of control animals 14 days after saline injection (Fig. 1A). In contrast, dramatic damages were observed in the retinas 14 days after injection, characterized by the presence of small dark blots, corresponding to degenerating profiles in the outer nuclear and cone and rod layer, throughout the whole retinal surface and the hyporeflection of retinal vessels in all retinas examined in this group (Fig. 1B). Pre-treatment with Theaphenon E, catechins mixture with EGCG, or EGCG alone reduced dramatically the degenerating profiles in the retina, as indicated by the number and distribution of the blots (Fig. 1D–F). Catechins without EGCG did not produce obvious reduction in these degenerating profiles (Fig. 1C). Quantitative analysis of the degenerative profiles showed that the number of blots increased progressively from 7 days to 14 days after injection of sodium iodate. Treatment with Theaphenon E, catechins mixture with EGCG or EGCG alone reduced significantly the number of dark blots on both 7 and 14 days after sodium iodate injection (*p* < 0.05), when compared to the vehicle-treated group (Fig. 1G). No statistically significant difference was observed among these groups. However, the decrease in rats treated with catechins mixture without EGCG was not significant (*p* > 0.05).

**Figure 2:**
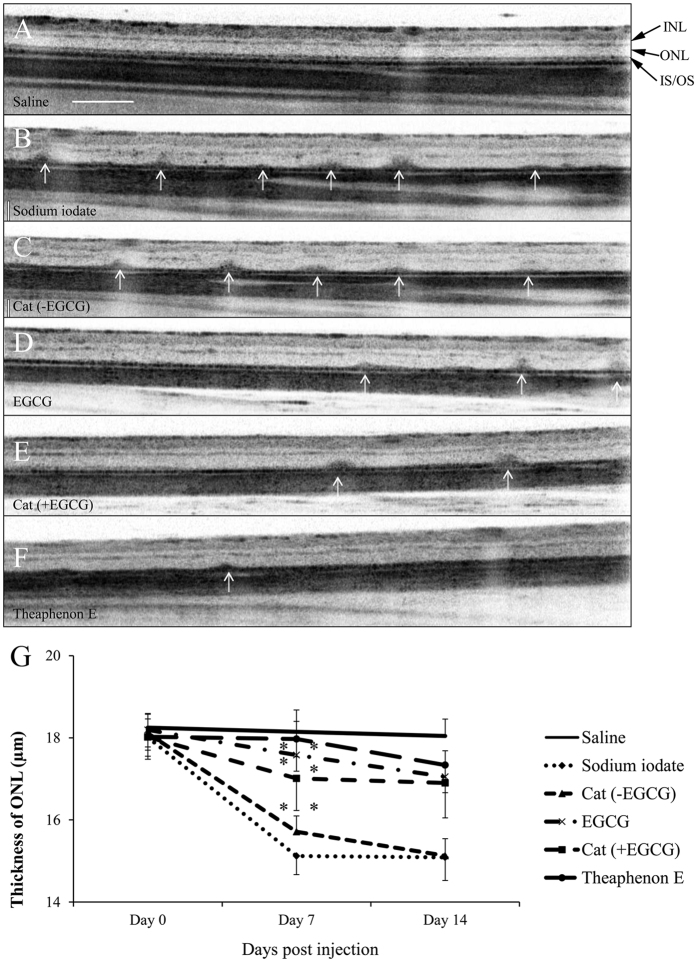
Optical coherence tomography analysis of sodium iodate-induced retinal degeneration with the treatment of GTE and its catechin constituents. Optical coherence tomography (OCT) images showing the cross-sectional retinal thickness of adult rats 14 days after injection of sodium iodate. (**A**) Saline control. (**B**) Injection of sodium iodate alone. Dark blots (white arrows) corresponding to degenerating profiles in the outer retinal layers were observed. (**C**) Treatment with catechin constituents (GC, EGC and EC) without EGCG [Cat (-EGCG)]. (**D**–**F**) The dark blots were highly reduced in the retina for the groups pre-treated with GTE Theaphenon E, or combination of catechin constituents containing EGCG. INL: inner nuclear layer; ONL: outer nuclear layer; IS/OS: inner segment and outer segment of photopigment layer. Scale bar is 200 μm. (**G**) Quantitative measurements of the thickness of outer nuclear layer in OCT images showed a substantial reduction 7 days after sodium iodate injection. The reduction was significant in the groups pre-treated with Theaphenon E, catechins mixture with EGCG, or EGCG alone, but not with catechins mixture without EGCG. The experimental data were compared with that of the sodium iodate group using Wilcoxon signed rank test, *p < 0.05, **p < 0.01. Data were presented as mean ± SEM.

OCT images showed that, starting from day 7 after sodium iodate injection, the outer nuclear layer (ONL) thickness was substantially reduced (Fig. 2A–F). This thinning of ONL was significantly reverted after treatment with Theaphenon E, catechins with EGCG, or EGCG alone (*p* < 0.01, compared to the vehicle-treated group; Fig. 2G). The effects were maintained on Day 14 after injection. Catechins mixture without EGCG did not show such protective effect to the ONL (*p* > 0.05).

### Histological analysis of sodium iodate-induced retinal degeneration with green tea catechin treatments

**Figure 3:**
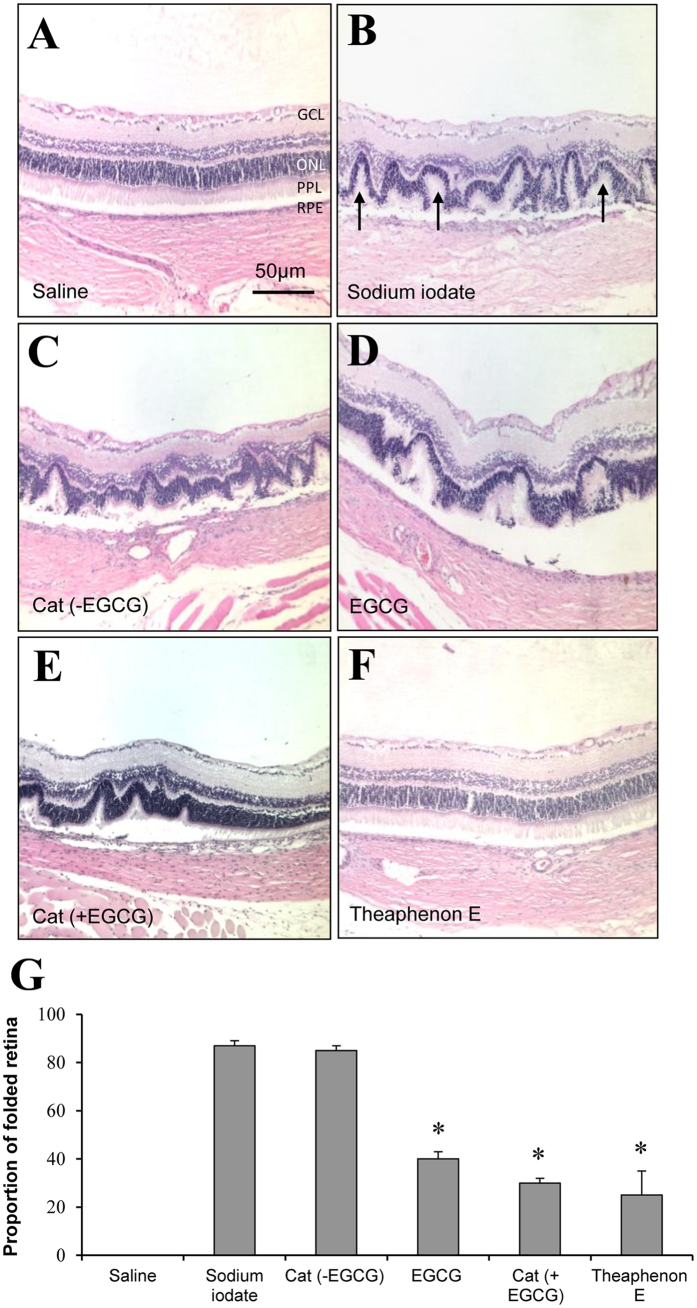
Catechins attenuated retinal damages induced by sodium iodate. Paraffin section of the rat retina stained with Hematoxylin and Eosin. All the images were taken 300 μm from the optic disk. (**A**) saline control; (**B**) extensive disruption was observed in the outer nuclear layer (ONL), photopigment layer (PPL) and retinal pigment epithelium (RPE) 14 days after sodium iodate injection. (**C**) Treatment with catechins without EGCG did not show obvious protection to the outer retinal layers. (**D**–**F**) The outer retinal lesions were alleviated with treatment of EGCG, catechins containing EGCG and Theaphenon E. Among these the GTE Theaphenon E seemed to provide the most potent protection to the retina. (**G**) Quantitative analyses of the lesion in ONL showed that the damages were ameliorated significantly with treatment of Theaphenon E, catechins with EGCG or EGCG alone. No obvious protection was found with catechins without EGCG. (*p < 0.05, compared with sodium iodate group). GCL: ganglion cell layer; IPL: inner plexiform layer; INL, inner nuclear cell layer; OPL: outer plexiform layer. Scale bar is 200 μm.

To validate the *in vivo* imaging results, degenerating profiles were measured in histological sections of the retina collected 14 days after sodium iodate injection. In the retina of sodium iodate injected rats, obvious damages were found in the RPE, cone and rod layer and ONL, forming series of irregular foldings in the outer regions of the retina (Fig. 3A,B). These damages were observed in all animals injected with sodium iodate and in all regions of the retina. Treatment with catechin mixture without EGCG did not produce any detectable changes to the lesions (Fig. 3C). However, EGCG, catechin mixture with EGCG and the GTE Theaphenon E substantially reduced these retinal damages (Fig. 3D–F).

This protective effect was supported by quantitative analysis of the damages in the outer retinal regions. The percentages of folded retina (total retinal length exhibiting irregular ONL vs total retinal length) in histological sections cutting across the optic disc were measured. More than 50% of the retinal folding was significantly reduced after pre-treatment with EGCG, catechin mixture with EGCG or Theaphenon E (*p* < 0.05). The protective effect is most potent for Theaphenon E (Fig. 3G). No obvious protection was observed in rats treated with catechin mixture without EGCG. These findings confirmed the live imaging results obtained by cSLO and OCT, demonstrating a potent protective effect of GTE to retinal degeneration induced by sodium iodate. EGCG is the most important constituent.

### Changes of oxidative stress markers

**Figure 4:**
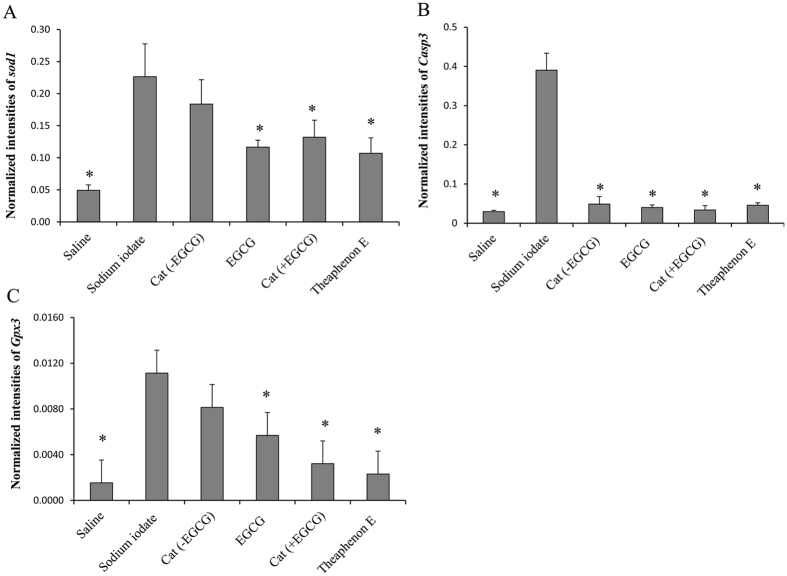
GTE and its catechin constituents suppressed expression of oxidative stress related genes in the retina. The expression of (**A**) superoxide dismutase (*Sod1*), (**B**) caspase 3 (*Casp3*) and (**C**) glutathione peroxidase (*Gpx3*) was highly elevated 24 hours after sodium iodate injection. Treatments with Theaphenon E, catechins with EGCG or EGCG alone reduced significantly levels of these genes. Catechins without EGCG did not show such reduction, except for caspase 3. *p < 0.05, Mann-Whitney U test, when compared with sodium iodate group. The data were presented as Mean ± SEM.

The effects of catechin treatment on the expression of oxidative stress related genes superoxide dismutase (*Sod1*), glutathione peroxidase (*Gpx3*) and caspase 3 (*Casp3*) was investigated in the retina 24 hours after sodium iodate injection. These genes were expressed at basal levels in the saline controls, but their expressions were increased significantly after sodium iodate injection (Fig. 4A–C). The elevated levels of *Sod1* and *Gpx3* were suppressed significantly after treatment with Theaphenon E, catechins with EGCG or EGCG (*p* < 0.05), when compared to the vehicle-treated group, but was not obvious for catechin mixture without EGCG (*p* > 0.05; Fig. 4A,C). The expression level of *Casp3* was reduced after treatment with Theaphenon E, EGCG or catechin mixture with or without ECGC (Fig. 4B), suggesting a potent anti-oxidative and anti-apoptotic effect of catechins against sodium iodate induced retinal damages.

**Figure 5:**
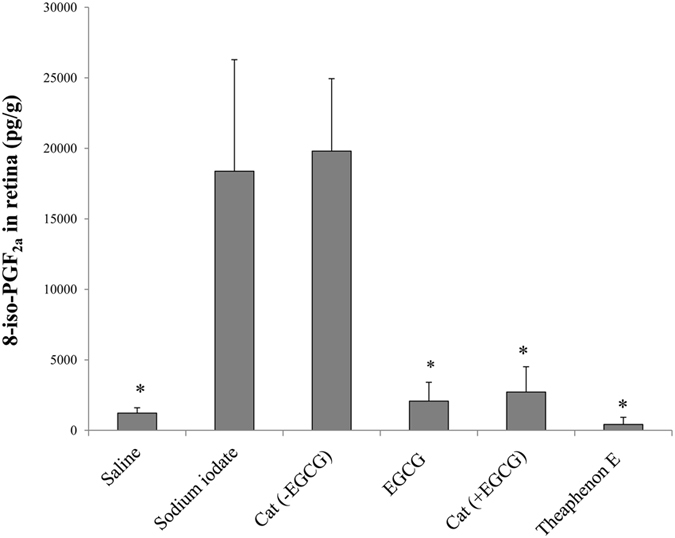
The oxidative stress marker 8-iso-PGF_2α_ was significantly reduced in retina after GTE treatment. Quantification of 8-iso-PGF_2α_ concentration 24 hours after sodium iodate injection showed a dramatic increase in the retina. These elevated productions were suppressed significantly in the groups pre-treated with Theaphenon E, combination of catechins containing EGCG, or EGCG alone, but not with catechins without EGCG. *p < 0.05, Mann-Whitney U test (Mean ± SEM).

The anti-oxidative properties of catechins were also investigated using 8-iso-prostanglandin F2α (8-iso-PGF_2α_) as an oxidative stress index. In retinas treated with 40 mg/kg sodium iodate, the level of 8-iso-PGF_2α_ was drastically increased for more than 10 folds in 24 hours (Fig. 5). Treatment with Theaphenon E, EGCG or catechin mixture with EGCG significantly reduced the 8-iso-PGF_2α_ level in the retina (*p* < 0.05, when compared to the vehicle-treated control). However, no significant reduction was observed after treatment with catechin mixture without EGCG (*p* > 0.05).

## Discussion

We have investigated the effects of GTE and its catechin constituents on retinal lesions generated by sodium iodate-induced oxidative stress in adult rat retina. Major findings include: i) *in vivo* imaging using cSLO and SD-OCT shows that sodium iodate induces a progressive and consistent damage to the retina; ii) histological examinations reveal a loss of RPE and disruption of cellular arrangement in the cone and rod layer and the outer nuclear layer; iii) these damages are alleviated effectively by the GTE Theaphenon E, and by its catechin constituents containing EGCG; iv) GTE and its catechin constituents suppress significantly the increases in *Sod1*, *Gpx3* and caspase 3, and reduce production of 8-iso-PGF_2α_ after sodium iodate insult. These findings demonstrate a potent protective effect of Theaphenon E and its constituent catechins in outer retinal degeneration caused by oxidative stress, and suggest a therapeutic use of GTE in ocular diseases, such as AMD, that involves degeneration of RPE and photoreceptor cells.

Sodium iodate is a known toxin to induce selective RPE damage by oxidative stress, and consequently retinal degeneration^17^,^18^. Sod1 is one of the major antioxidant enzymes that play a crucial role in scavenging superoxide. Gpx3 is shown to remove free radicals and superoxides by means of glutathione^19^. In this study, we observed a significantly elevated expression of the Sod1 and Gpx3 in the retina after sodium iodate injection, suggesting an increased utilization for scavenging reactive oxygen species during the insult. These oxidative damages are ameliorated by the catechins, which contain ortho-hydroxyl group in the B-ring and galloyl moiety in the C-ring that react directly with superoxide and reduce formation of H_2_O_2_^20^. The anti-oxidative effect of catechins is further shown by measuring the level of 8-isoprostane, a marker of oxidative stress. This molecule is a well characterized member of the F_2_-isoprostanes family, a group of stable PGF_2α_ isomers derived from non-enzymatic oxidation of arachidonic acid independent of cyclooxygenase activity^21^. In the current study, the surge of 8-isoprostane level due to oxidative stress was dramatically decreased after treatment with GTE or its catechin constituents, giving direct evidences of potent anti-oxidative effects of these compounds. Moreover, analyses of different combinations of catechin constituents showed that the anti-oxidative effects are drastically reduced if EGCG is excluded. Therefore, EGCG is the most active constituent that contributes to the anti-oxidative activities of GTE. However, it remains to be determined if other catechin constituents exert more potent anti-oxidative effects at concentrations higher than those used in this study.

Previous reports have demonstrated that apoptosis plays a pivotal role in photoreceptor degeneration in sodium iodate-treated animals, and that GTE and catechins possess anti-apoptotic effects. This has been shown in rats with photoreceptor degeneration, in which the number of TUNEL-labeled nuclei was significantly reduced in catechins-treated eyes than in untreated eyes^12^. Results in the current study also support the anti-apoptotic action of catechins, as caspase-3 is highly suppressed after GTE and catechin treatments, likely caused by a reduction in anti-oxidative stress.

The neuroprotective effects of GTE are partly contributed by the accessibility of various catechin constituents to the ocular tissues. Our recent studies have shown that oral administration of GTE can reach the retinal tissue in sufficient amount to protect it from detrimental insult of oxidative stress^22^,^23^. In our previous study, when another GTE, Sunphenon DCF-1, is orally administered at the dose of 550 mg/kg to the rats, various catechin constituents are detected in various tissues and compartments of the eye, and the peak concentrations are reached from 0.5 to 12.2 hr^23^. GC is predominant in vitreous humor, whereas EGC and EC are enriched in the retina. These catechins are sustained at high levels in the retina. However, penetration level of EGCG is not particularly high^23^. In Theaphenon E, the amount of individual catechin constituents is higher than those in Sunphenon DCF-1, especially EGCG, resulting in increased accumulation of catechins in various ocular tissues. A mixture of catechins in GTE, such as that in Theaphenon E, is better than purified catechins, for example EGCG, because of synergic effects of various catechin constituents on anti-oxidation and bioavailability. A comparison of neuroprotective effects in our live imaging studies and histological sections shows that Theaphenon E and combination of catechins with EGCG are more potent than EGCG alone, suggesting additional and synergistic effects among major and minor constituents of catechins in GTE. It would be important to determine the optimal composition that generates the most potent protecting effect against oxidative stress-induced retinal degeneration.

In summary, the results from our *in vivo* and *in vitro* investigations show that oral intake of GTE or its catechin constituents attenuates sodium iodate-induced retinal degeneration in rats by rescuing RPE loss and disruption of ONL through a suppression of oxidative stress. Our findings suggest that GTE and its catechin constituents can serve as potent therapeutic agents to retinal degeneration, and support the notion that daily consumption of GTE is beneficial to patients suffering from AMD and retinal dystrophies where oxidative stress is implicated^24^.

## Methods

### Animals

All rats were treated according to Guidelines of the Association for Research in Vision and Ophthalmology (ARVO) Statement on Use of Animals in Ophthalmic and Vision Research. Experimental protocol in this study was approved by the Animal Experimentation Ethics Committee of the Chinese University of Hong Kong. Adult Sprague-Dawley rats, weighing 200 g to 250 g, were obtained from the Laboratory Animal Service Center of the Chinese University of Hong Kong. Animals were housed in standard condition, maintained at 22 ± 1 °C, 40 ± 10% humidity and with 12:12 hour dark-light cycle. Standard rodent chow and water were provided *ad libitum*. For each experimental group, 5 rats were used for the experiments.

### Sodium iodate and drug administration

Sodium iodate (Sigma-Aldrich, St. Louis, MO) was dissolved in sterile normal saline at a stock concentration of 4% (w/v). The rats were anesthetized by intraperitoneal (i.p.) injection of ketamine (35 mg/kg; Ketaset; Fort Dodge Animal Health, Fort Dodge, IA) and xylazine (5 mg/kg; TranquiVed; Vedco, Inc., St. Joseph, MO). Intravenous injection (i.v.) of 40 mg/kg sodium iodate was given via the tail vein. This dosage was shown to be optimal concentration that induces extensive outer retinal lesion throughout the whole retinal surface without causing obvious toxic effects to other major organs^13^. The control group was the rats injected i.v. with the same volume of normal saline. The catechin constituents EGCG, EC, GC, EGC (purchased from Chengdu Biopurity Phytochemical, China) and the GTE Theaphenon E (provided generously by Dr. Yukihiko Hara, Department of Environmental Physiology, Shimane University Faculty of Medicine, Japan) were suspended in distilled water.

To determine the most active components of GTE, we studied the neuroprotective effect of GTE and its major constituents. The dosages of catechins tested were in accord to the proportion of each constituent in the GTE Theaphenon E (EGCG: 70.53%, EGC: 4.61%, EC: 3.88%, GC: 0.64%)^23^. The dosages of catechins in the experimental groups are as follows: i) Theaphenon E - 550 mg/kg, which has been shown in our earlier study to be the optimal dose that produces potent anti-inflammatory effects against acute uveitis in the rat without causing obvious toxic actions to major organs^22^, ii) EGCG - 387.8 mg/kg; iii) catechins mixture with EGCG - 438.0 mg/kg, contains EGCG (387.8 mg/kg), GC (3.53 mg/kg), EGC (25.4 mg/kg) and EC (21.4 mg/kg); iv) catechins mixture without EGCG - 50.3 mg/kg, with GC (3.53 mg/kg), EGC (25.4 mg/kg) and EC (21.4 mg/kg). The catechins were fed intragastrically twice at 12 hours and 1 hour before sodium iodate injection. Each experimental group and control group consisted of five animals.

### Confocal scanning laser ophthalmoscopy and spectral-domain optical coherence tomography

cSLO and spectral-domain OCT (HRA-II; Heidelberg Engineering GmbH, Dossenheim, Germany) were used for *in vivo* imaging of the retina of live rats. The procedures were as described in our earlier report^13^. In brief, an illuminating wavelength of 870 μm with a bandwidth of 50 μm was used as a broadband light source. A dual-beam simultaneous imaging with an infrared cSLO provides a planar visualization of the retina. The scan rate of the cSLO was 16 frames per second with eye-tracking activated. Fifteen images at the same retinal location at the same focal depth were captured. These images were averaged automatically by the built-in software to augment the signal-to-noise ratio and simultaneously displayed on a computer screen^25^. The Spectralis OCT was modified according to the technical advice from the manufacturer for OCT imaging of rats. Retinal photographs and OCT images could be simultaneously captured on the exact retinal locus, which ensures the high quality of OCT imaging. In each retina, 4 different square regions, each from the superotemporal, inferotemporal, inferonasal and superonasal quadrant, around the optic nerve head were scanned separately by the volume scan protocol, which consists of 19 evenly distributed B scans (1024 A scans for each B scan) covering a 20° × 15° area of the retina^26^. The images were captured before (Baseline) and after sodium iodate injection (on Day 7 and 14). Prior to imaging, rats were anesthetized and pupils were dilated by topical 1% tropicamide. During imaging, media clarity was maintained with sterile saline to the cornea. The number of blots, which correspond to the degenerating profiles in outer regions of the retina, was analyzed by Photoshop (11.0; Adobe Systems Incorporated, San Jose, CA)^16^. In each retina, 4 different square regions (400 μm × 400 μm) with clear visualization of the blots were analyzed before and after injection by a blinded observer.

### Histological assessment of the retina

On post-injection day 14, the rats were terminated with overdose of sodium pentobarbital (20%, W/V) and perfused with phosphate buffered saline (PBS) followed by 4% paraformaldehyde (Sigma-Aldrich) in PBS at pH 7.4. Eyes were removed and further fixed in 10% formalin before paraffin embedding. Five micrometer sections of the eye were collected at the pupil-optic nerve position. The sections were stained with Hematoxylin and Eosin, and images were captured by a light microscope (DMRB; Leica, Germany) connected to a Spot digital camera (Diagnostic Instrument Inc, USA).

### Assay for 8-Iso-PGF_2α_ in retina

The 8-Iso-PGF_2α_ assay was based on our previously published protocol^21^. Briefly, on post-injection day 1, the rats were sacrificed with overdose of sodium pentobarbital. The retinas were dissected and homogenized in ice-cold Folch solution. The homogenate was washed in 0.5 ml 0.9% sodium chloride to remove water-soluble compounds and protein residues. After centrifugation, the organic layer was collected and dried under nitrogen. The organic residues of the tissues were hydrolyzed by potassium hydroxide (15%) for 30 min at 37 °C. After acidification by hydrochloric acid to pH 3, 8-iso-PGF_2α_ was extracted by C18 and silica Sep-Pak, derivatized with pentafluorobenzyl bromide (PFBB) and purified by thin layer chromatography (TLC). The isoprostane residues were scraped out, extracted by ethyl acetate and derivatized by N,O-bis-(trimethylsilyl)-trifluoroacetamide (BSTFA), then dried and dissolved in dodecane for GC-NCI-MS analysis.

### Gene expression analysis

Total RNA was isolated and extracted by TRIzol reagent (Invitrogen) followed by the RNeasy extraction kit according to the manufacturer’s protocol (Qiagen, Germany). Equal amount of total RNA (2 μg) from each sample was reverse-transcribed using SuperScript III cDNA synthesis kit (Invitrogen). Expression of oxidative stress-related genes was evaluated by the SYBR Green PCR (Roche) using the LightCycler real-time PCR instrument (Roche). Gene-specific primers for all tested genes were listed in Table 1. Thermal cycling conditions included an initial denaturation step at 95 °C for 3 min followed by 45 cycles at 95 °C for 30 sec, and an annealing temperature at 60 °C for 30 sec. All samples were run in triplicate, and each well contained 20 μl as final volume, including 1 μl of cDNA, 10 μM gene-specific primers and 2x SYBR Green I (Roche). *Gapdh* was used as housekeeping gene. Negative controls for gene expression were those without RNA in the reverse transcription reaction and without cDNA in the SYBR green PCR reaction. The mathematical method described by Pfaffl was used to evaluate the relative expression as compared with *Gapdh*^27^.

### Statistical analysis

All data were analyzed by non-parametric tests, Mann-Whitney U-test and Wilcoxon signed rank test, were used to compare the mean of each experimental group with that of negative control group. Data were expressed as mean ± standard error of the mean (SEM). All analyses were performed using PASW Statistics 18 (SPSS Science, Chicago, IL), and *p* < 0.05 was considered as statistical significance.

## Additional Information

**How to cite this article**: Yang, Y. *et al*. Green tea catechins are potent anti-oxidants that ameliorate sodium iodate-induced retinal degeneration in rats. *Sci. Rep.* **6**, 29546; doi: 10.1038/srep29546 (2016).

## Figures and Tables

**Table 1:** Primer Sequences of oxidative stress markers for gene expression analysis.

**Gene**		**Sequence (5′** **>** **3′)**	**Length (nt)**	**Annealing Tm (°C)**	**Amplicon size (bp)**	**Reference**
*Gadph*	F:	GTGCCAGCCTCGTCTCATA	19	60	190	NM_017008.3
	R:	GTTGAACTTGCCGTGGGTAG	20			
*Sod1*	F:	GGATGAAGAGAGGCATGTTGG	21	60	122	NM_017050.1
	R:	TACGGCCAATGATGGAATGC	20			
*Gpx3*	F:	TTCGGACACCTCAGACGG	18	60	149	NM_022525.3
	R:	GGCAGTCTGTCTTGGACTTC	20			
*Casp3*	F:	AGTCTGACTGGAAAGCCGAA	21	60	122	NM_017050.1
	R:	ATAGTAACCGGGTGCGGTAG	20			

## References

[b1] Klein R., Peto T., Bird A. & Vannewkirk M. R. The epidemiology of age-related macular degeneration. Am. J. Ophthalmol. 137, 486–495 (2004). 15013873 10.1016/j.ajo.2003.11.069

[b2] Wong W. L. . Global prevalence of age-related macular degeneration and disease burden projection for 2020 and 2040: A systematic review and meta-analysis. *Lancet Glob*. *Health* 2, e106–116 (2014). 25104651 10.1016/S2214-109X(13)70145-1

[b3] Jager R. D., Mieler W. F. & Miller J. W. Age-related macular degeneration. N. Engl. J. Med. 358, 2606–2617 (2008). 18550876 10.1056/NEJMra0801537

[b4] Ng T. K. . Interactive expressions of HtrA1 and VEGF in human vitreous humors and fetal RPE cells. Invest. Ophthalmol. Vis. Sci. 52, 3706–3712 (2011). 21310902 10.1167/iovs.10-6773

[b5] Ng T. K., Liang X. Y. & Pang C. P. HTRA1 in age-related macular degeneration. Asia Pac. J. Ophthalmol . 1, 51–63 (2012). 10.1097/APO.0b013e31823e57fe 26107018

[b6] de Jong P. T. Age-related macular degeneration. N. Engl. J. Med. 355, 1474–1485 (2006). 17021323 10.1056/NEJMra062326

[b7] Zarbin M. A. Current concepts in the pathogenesis of age-related macular degeneration. Arch. Ophthalmol. 122, 598–614 (2004). 15078679 10.1001/archopht.122.4.598

[b8] Zhang B., Safa R., Rusciano D. & Osborne N. N. Epigallocatechin gallate, an active ingredient from green tea, attenuates damaging influences to the retina caused by ischemia/reperfusion. Brain Res. 1159, 40–53 (2007). 17573045 10.1016/j.brainres.2007.05.029

[b9] Chu K. O. . Pharmacokinetic studies of green tea catechins in maternal plasma and fetuses in rats. J. Pharm. Sci. 95, 1372–1381 (2006). 16625654 10.1002/jps.20594

[b10] Cao G. . EGCG protects against UVB-induced apoptosis via oxidative stress and the JNK1/c-Jun pathway in ARPE19 cells. Mol. Med. Rep . 5, 54–59 (2012). 21909619 10.3892/mmr.2011.582

[b11] Costa B. L., Fawcett R., Li G. Y., Safa R. & Osborne N. N. Orally administered epigallocatechin gallate attenuates light-induced photoreceptor damage. Brain Res. Bull. 76, 412–423 (2008). 18502318 10.1016/j.brainresbull.2008.01.022

[b12] Zhang B. & Osborne N. N. Oxidative-induced retinal degeneration is attenuated by epigallocatechin gallate. Brain Res. 1124, 176–187 (2006). 17084820 10.1016/j.brainres.2006.09.067

[b13] Yang Y. . Assessing sodium iodate-induced outer retinal changes in rats using confocal scanning laser ophthalmoscopy and optical coherence tomography. Invest. Ophthalmol. Vis. Sci. 55, 1696–1705 (2014). 24526437 10.1167/iovs.13-12477

[b14] Amirpour N. . Differentiation of human embryonic stem cell-derived retinal progenitors into retinal cells by Sonic hedgehog and/or retinal pigmented epithelium and transplantation into the subretinal space of sodium iodate-injected rabbits. Stem Cells Dev. 21, 42–53 (2012). 21456900 10.1089/scd.2011.0073

[b15] Gong L., Wu Q., Song B., Lu B. & Zhang Y. Differentiation of rat mesenchymal stem cells transplanted into the subretinal space of sodium iodate-injected rats. Clin. Experiment Ophthalmol. 36, 666–671 (2008). 18983552 10.1111/j.1442-9071.2008.01857.x

[b16] Ohtaka K. . Protective effect of hepatocyte growth factor against degeneration of the retinal pigment epithelium and photoreceptor in sodium iodate-injected rats. Curr. Eye Res. 31, 347–355 (2006). 16603468 10.1080/02713680600629797

[b17] Kiuchi K., Yoshizawa K., Shikata N., Moriguchi K. & Tsubura A. Morphologic characteristics of retinal degeneration induced by sodium iodate in mice. Curr. Eye Res. 25, 373–379 (2002). 12789545 10.1076/ceyr.25.6.373.14227

[b18] Zhao C. . mTOR-mediated dedifferentiation of the retinal pigment epithelium initiates photoreceptor degeneration in mice. J. Clin. Invest. 121, 369–383 (2011). 21135502 10.1172/JCI44303 PMC3007156

[b19] Gandhi S. & Abramov A. Y. Mechanism of oxidative stress in neurodegeneration. *Oxid. Med. Cell Longev*. 2012, 428010 (2012). 22685618 10.1155/2012/428010 PMC3362933

[b20] Chu K. O. . Effects of EGCG content in green tea extract on pharmacokinetics, oxidative status and expression of inflammatory and apoptotic genes in the rat ocular tissues. J. Nutr. Biochem. 26, 1357–1367 (2015). 26362107 10.1016/j.jnutbio.2015.07.001

[b21] Wang C. C. . Tea epigallocatechin-3-gallate increases 8-isoprostane level and induces caudal regression in developing rat embryos. Free Radic. Biol. Med. 43, 519–527 (2007). 17640562 10.1016/j.freeradbiomed.2007.04.034

[b22] Qin Y. J. . Green tea extract treatment alleviates ocular inflammation in a rat model of endotoxin-induced uveitis. PLoS One 9, e103995 (2014). 25093862 10.1371/journal.pone.0103995 PMC4122397

[b23] Chu K. O. . Green tea catechins and their oxidative protection in the rat eye. *J. Agric. Food Chem*. 58, 1523–1534 (2010). 20085274 10.1021/jf9032602

[b24] Narotzki B., Reznick A. Z., Navot-Mintzer D., Dagan B. & Levy Y. Green tea and vitamin E enhance exercise-induced benefits in body composition, glucose hmomeostasis, and antioxidant status in elderly men and women. J. Am. Coll. Nutr. 32, 31–40 (2013). 24015697 10.1080/07315724.2013.767661

[b25] Li Z. W. . Tracking dendritic shrinkage of retinal ganglion cells after acute elevation of intraocular pressure. Invest. Ophthalmol. Vis. Sci. 52, 7205–7212 (2011). 21775662 10.1167/iovs.10-6868

[b26] Leung C. K. . Comparison of macular thickness measurements between time domain and spectral domain optical coherence tomography. Invest. Ophthalmol. Vis. Sci. 49, 4893–4897 (2008). 18450592 10.1167/iovs.07-1326

[b27] Pfaffl M. W. A new mathematical model for relative quantification in real-time RT-PCR. Nucleic Acids Res. 29, e45 (2001). 11328886 10.1093/nar/29.9.e45 PMC55695

